# QuEChERS-GC-MS/MS Analysis of Multi-Class Pesticide Residues in Tropical Agricultural Soils

**DOI:** 10.3390/molecules31152564

**Published:** 2026-07-23

**Authors:** Diego Alejandro Riaño-Herrera, Laura Herrera-Paiva, Julien Gwendal Chenet, Alberto Uribe-Jongbloed, Diana Angélica Varela-Martínez, Miguel Ángel González-Curbelo

**Affiliations:** 1 Departamento de Ingeniería Ambiental y Energías, Facultad de Ingeniería, Universidad EAN, Calle 79 No. 11-45, Bogotá 111321, Colombia; dariano@universidadean.edu.co (D.A.R.-H.); lherrera1451@universidadean.edu.co (L.H.-P.); jgchenet@universidadean.edu.co (J.G.C.); auribejo@universidadean.edu.co (A.U.-J.); 2 Departamento de Ciencias Básicas, Facultad de Ingeniería, Universidad EAN, Calle 79 No. 11-45, Bogotá 111321, Colombia; davarela@universidadean.edu.co

**Keywords:** sample preparation, method validation, matrix effects, pesticide residues, soil analysis, QuEChERS

## Abstract

The determination of pesticide residues at trace levels in complex environmental matrices requires robust and reliable analytical methodologies. In this study, a multi-residue method for determining 48 pesticides across different chemical classes in agricultural soils was developed and validated using a modified QuEChERS extraction followed by GC-MS/MS analysis. The method exhibited good linearity (R^2^ ≥ 0.9871) over a concentration range of 5–600 µg/kg. Matrix effects were observed for several compounds, requiring matrix-matched calibration for accurate quantification. Recoveries fulfilled the recommended 70−120% criterion in 91.0% of the evaluated cases, while more than 98% of the relative standard deviation values were ≤20%, demonstrating satisfactory overall accuracy and precision according to SANTE/2020/12830 (Rev. 2) guidelines. The validated method was subsequently applied to tropical agricultural soils from Puerto Carreño (Colombian Orinoquia), which exhibited predominantly sandy textures (>80% sand), slightly acidic pH (5.2−5.7), low total organic carbon (0.1−0.8%), and low cation exchange capacity (5.6−8.1 meq/100 g). No pesticide residues were detected in the analyzed samples. The proposed method provides a reliable analytical framework for multi-class pesticide determination in tropical agricultural soils and contributes to the application of QuEChERS-based methodologies in underrepresented tropical soil matrices.

## 1. Introduction

The widespread use of pesticides in modern agriculture has led to increasing concern regarding their persistence and distribution in the environment [1]. These compounds can occur at trace levels, making their reliable determination a central objective in analytical chemistry [2,3]. In addition, pesticides encompass a wide range of physicochemical properties, including differences in polarity, volatility, and thermal stability, which pose significant challenges for their simultaneous extraction, separation, and detection within a single analytical workflow [4]. In this context, the development of multi-residue methodologies capable of detecting diverse pesticide classes with high sensitivity and selectivity has become essential.

Soil represents a primary compartment for pesticide deposition due to its direct exposure during agricultural applications, but these compounds may also reach the soil through processes such as drift, volatilization, and atmospheric deposition [5,6]. As a result, residues can be detected even in areas without a documented history of intensive use. In addition, the persistence of certain pesticides, associated with their chemical stability and resistance to degradation, allows them to remain in soils over extended periods [6]. This behavior, combined with the continuous input of different compounds, may lead to the presence of complex residue mixtures at low concentration levels, posing additional challenges for their accurate identification and quantification.

At the same time, soil constitutes a complex analytical matrix characterized by heterogeneous composition and variable physicochemical properties. Parameters such as texture, pH, organic matter content, and cation exchange capacity influence pesticide behavior, including their distribution and persistence [7,8]. From an analytical perspective, these properties can affect extraction efficiency and instrumental response [9], which may ultimately impact the reliability of multi-residue methods across different soil types. In particular, the co-extraction of endogenous matrix components can lead to matrix effects (MEs) during chromatographic analysis, resulting in signal suppression or enhancement and affecting quantitative accuracy [10,11]. These effects are especially relevant in mass spectrometry-based techniques, where interactions between analytes and co-eluting substances can alter ionization efficiency [10]. Consequently, soil analysis requires methodologies that are not only sensitive and selective but also adaptable to variations in matrix composition, ensuring consistent analytical performance under diverse conditions.

Multi-residue analytical approaches have become increasingly relevant for pesticide determination, allowing the simultaneous analysis of compounds from different chemical families. Among these, the QuEChERS (Quick, Easy, Cheap, Effective, Rugged, and Safe) method has been widely adopted due to its operational simplicity, reduced solvent consumption, and adaptability to different analytical scenarios [12]. Its workflow, based on acetonitrile (ACN) extraction followed by salt-induced partitioning and dispersive solid-phase extraction (dSPE) clean-up, enables efficient extraction of multiple compounds while minimizing sample handling steps [13,14]. These features make QuEChERS particularly suitable for high-throughput analysis and routine applications. Furthermore, its combination with gas chromatography-tandem mass spectrometry (GC-MS/MS) provides a robust analytical platform for the determination of semi-volatile pesticides with high selectivity and sensitivity [15].

Despite these advantages, the application of QuEChERS-based methodologies to soil matrices requires consideration of matrix-specific characteristics [16]. Adjustments in extraction procedures or clean-up strategies are often necessary to ensure adequate analytical performance [17]. In this regard, the joint consideration of soil physicochemical properties and method validation can offer useful contextual information when evaluating analytical results. While numerous studies have reported pesticide residue analysis in soils using QuEChERS-based approaches [16,17], tropical soils remain comparatively underrepresented. Expanding analytical studies to include these soil types contributes to a more comprehensive evaluation of method applicability across diverse environmental conditions and helps address the limited analytical information currently available for tropical agricultural soils.

This study aimed to validate and apply a modified QuEChERS-GC-MS/MS analytical methodology for the determination of pesticides belonging to different chemical classes in tropical agricultural soils from the Colombian Orinoquia. Particular emphasis was placed on evaluating the applicability of the proposed analytical strategy in a tropical soil matrix that remains underrepresented in the current literature. In addition, selected physicochemical properties were determined to characterize the soil matrices in which the method was validated and applied. This integrated approach contributes to the assessment of method performance in tropical soils and supports the applicability of QuEChERS-based multi-residue methodologies for pesticide monitoring in underrepresented agricultural environments.

## 2. Results and Discussion

### 2.1. Soil Physicochemical Characteristics

Physicochemical characterization revealed that the investigated soils shared similar textural and chemical properties. Parameters such as soil texture, pH, total organic carbon, and cation exchange capacity are recognized as key factors influencing pesticide retention, mobility, and analytical behavior, thereby affecting MEs and overall method performance during residue analysis [9,17]. The results obtained for the studied soils are summarized in Table 1.

According to the textural classification determined by the modified Bouyoucos method [18], one sampling location corresponded to a sandy loam soil, whereas the remaining locations were classified as sandy soils. Overall, these soils featured high sand contents (>80%) and low clay contents (<3%). Such coarse-textured soils generally exhibit lower sorption capacities for organic contaminants than fine-textured soils because of their reduced specific surface area and lower abundance of reactive mineral phases [7,8].

The soils exhibited slightly acidic conditions, with pH values ranging from 5.2 to 5.7. Soil pH is recognized as an important factor influencing the ionization state, degradation behavior, and sorption of pesticides, thereby affecting their environmental fate and analytical recovery depending on the physicochemical properties of individual compounds [19,20]. Total organic carbon contents were consistently low, ranging from 0.1 to 0.8%, whereas cation exchange capacity values varied between 5.6 and 8.1 meq/100 g. These results are consistent with the predominance of sandy materials and the highly weathered nature of tropical savanna soils reported for the Colombian Orinoquia region [21]. In fact, low total organic carbon and cation exchange capacity values are characteristic of highly weathered tropical soils with limited contents of organic matter and reactive sorption sites [7,8]. These properties are commonly considered relevant descriptors in studies addressing the occurrence and behavior of pesticides in soils because they influence pesticide retention and transport processes [19,20,22].

Overall, the investigated soils constituted a relatively homogeneous group from a physicochemical perspective. The narrow ranges observed for texture, pH, total organic carbon, and cation exchange capacity support the use of one sampling location as the source of the validation matrix for matrix-matched calibration and recovery experiments. Under these conditions, the selected validation matrix was considered representative of the investigated soils and suitable for method validation purposes within the study area. At the same time, the analysis of individual field samples preserved the spatial resolution required for the environmental assessment of pesticide residues across the study area.

### 2.2. Performance of the QuEChERS-GC-MS/MS Method

The performance of the modified QuEChERS-GC-MS/MS methodology was evaluated according to the criteria established in the SANTE/2020/12830 guidance document [23]. Method validation included the assessment of linearity, MEs, recovery, and precision. Selectivity was confirmed, as no significant interferences were observed at the retention times of the target analytes under the optimized chromatographic conditions.

Several complementary strategies were implemented to mitigate matrix-related interferences during GC-MS/MS analysis. The QuEChERS clean-up incorporated primary secondary amine (PSA), octadecylsilane (C18), and Florisil as dSPE sorbents. Florisil has been reported as an effective sorbent for the removal of polar and moderately polar matrix constituents through hydrogen-bonding, dipole–dipole, and Lewis acid-base interactions, thereby complementing the clean-up mechanisms provided by PSA and C18 [24,25]. The combined use of these sorbents was therefore intended to improve extract cleanliness and reduce the amount of co-extracted matrix components reaching the chromatographic system. In addition, analyte protectants (APs) were incorporated into the final extracts prior to injection. These compounds have been incorporated in GC-based pesticide analysis to reduce analyte degradation, minimize adsorption at active sites within the injection port and chromatographic system, and improve signal stability, particularly in complex matrices [26,27]. A deuterated internal standard (IS) (atrazine-d5) was further employed to compensate for variations associated with sample preparation and instrumental analysis. Nevertheless, the use of a single isotopically labelled IS may not fully account for analyte-specific extraction efficiencies, MEs, and instrumental responses across a chemically diverse group of pesticides, representing an inherent limitation of broad-scope multiresidue methods. To further control residual matrix-induced effects, matrix-matched calibration was applied to improve quantification accuracy. The combined application of APs, atrazine-d5, and matrix-matched calibration contributed to the overall robustness of the method despite the complexity of the soil matrix.

Matrix-matched calibration curves were constructed over the concentration range of 5–600 µg/kg. The lowest calibration level established for all analytes was 5 µg/kg. Good linearity was observed for all target pesticides, with coefficients of determination (R^2^) exceeding 0.9871. Detailed matrix-matched and solvent-based calibration data, including ME percentages, are provided in Table S1 of the Supplementary Material.

**Figure 1:**
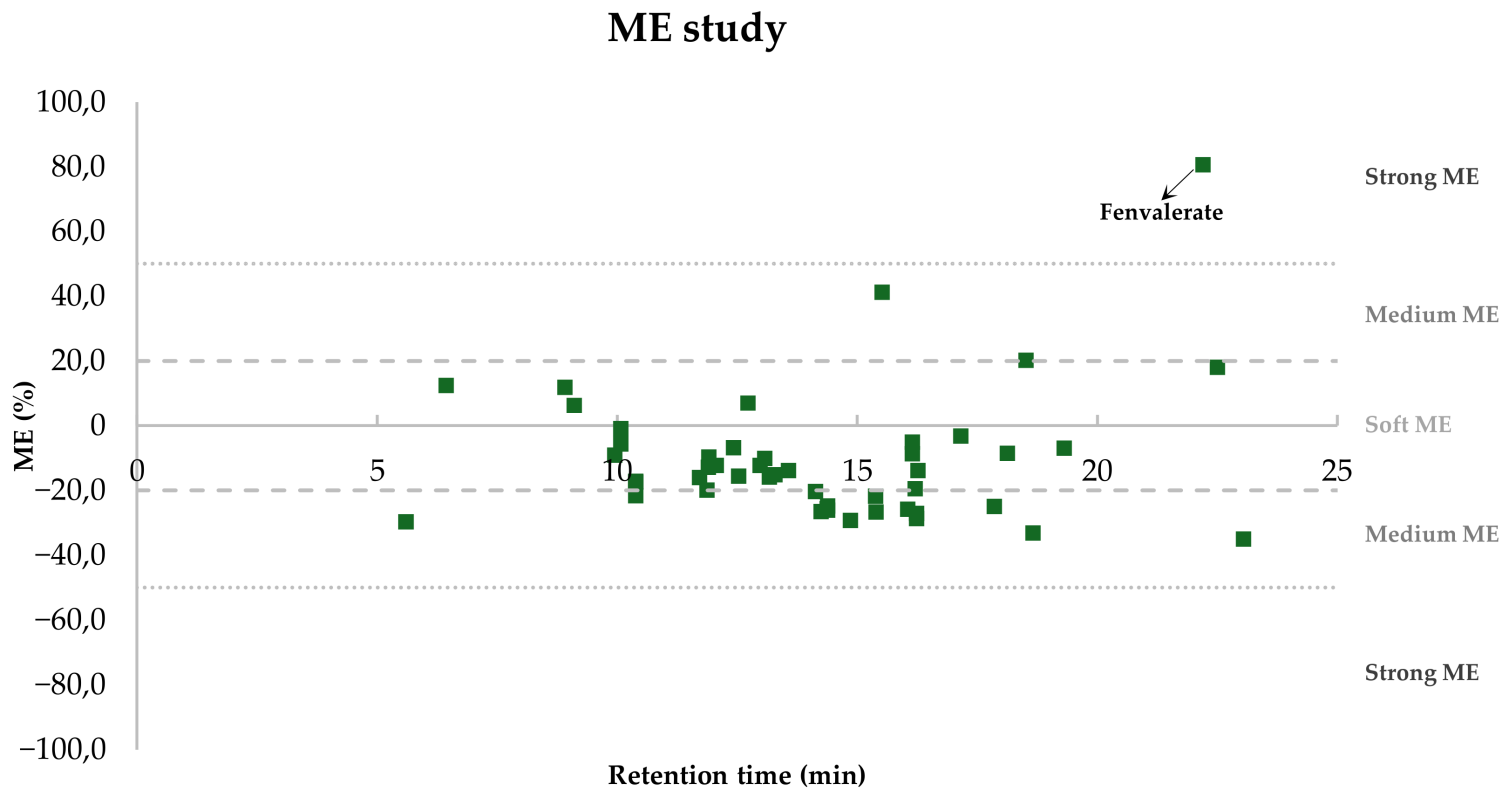
Distribution of the MEs versus retention times (min) for the 48 target pesticides.

MEs were evaluated by comparing the slopes of matrix-matched and solvent-based calibration curves. As shown in Figure 1, matrix-induced signal variations were observed for several compounds. Based on commonly accepted classification criteria [28], soft MEs (−20% to 20%) were observed for 29 pesticides (60.4%), medium MEs (−20% to −50% or 20% to 50%) were observed for 18 pesticides (37.5%), and a strong ME (>50% or <−50%) was observed for only one pesticide (2.1%), namely fenvalerate. Although MEs were observed for several analytes, their distribution was predominantly within the soft and medium ranges, suggesting that the combined clean-up and signal compensation strategy was generally effective in reducing, although not fully eliminating, matrix-induced signal variability. Consequently, matrix-matched calibration was used for both recovery experiments and quantification of field samples to ensure accurate determination.

**Figure 2:**
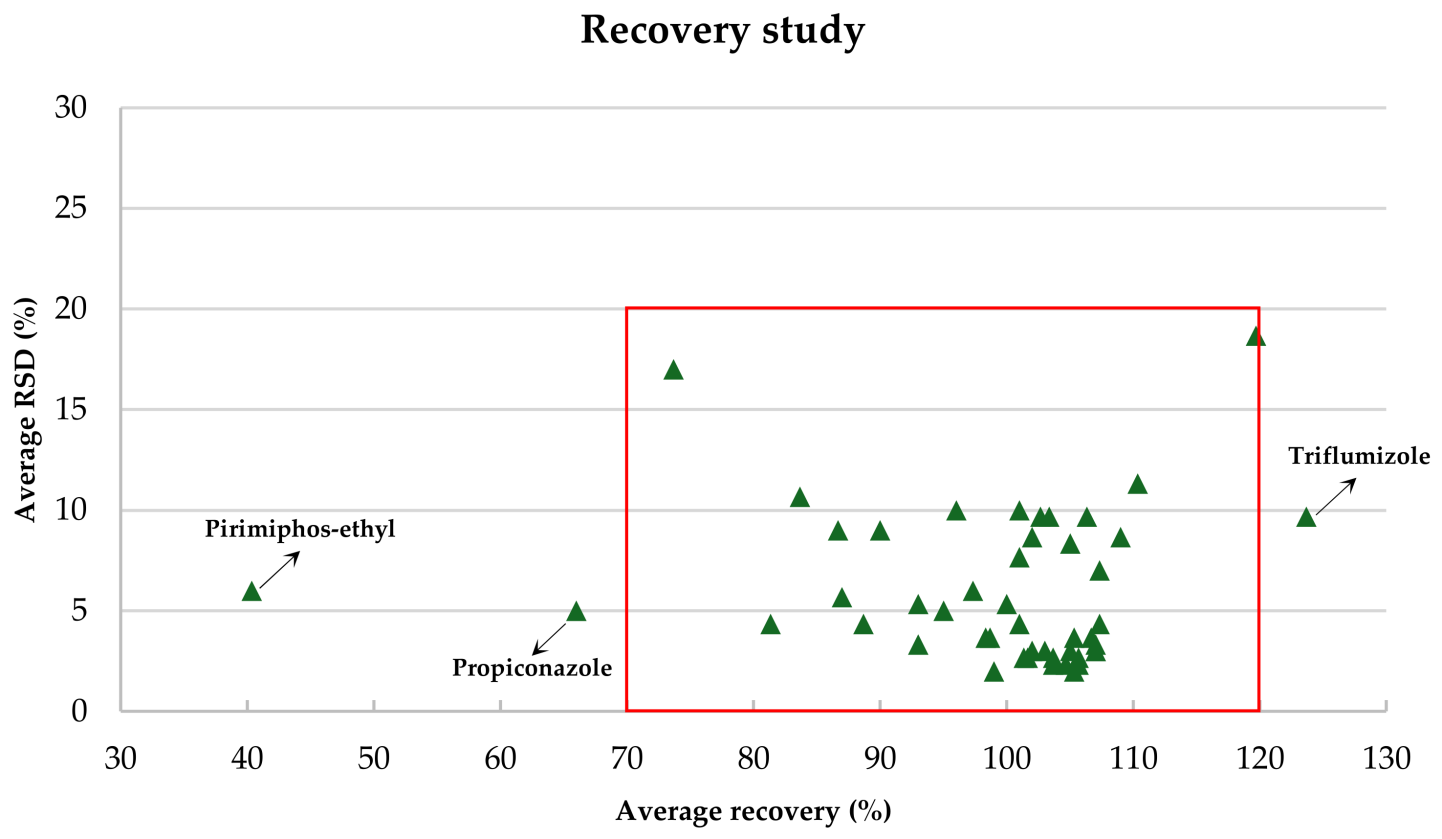
Average recoveries for the 48 pesticides after spiking 50 µg/kg, 200 µg/kg, and 400 µg/kg (*n* = 15) in the validation soil sample. The number of analytes within the acceptable recovery (70–120%) and RSD (<20%) ranges, together with those outside these criteria, are indicated.

Method accuracy and precision were assessed through recovery experiments performed at three fortification levels (50, 200, and 400 µg/kg). The lowest fortification level corresponded to the target method limit of quantification (LOQ) of 50 µg/kg. Representative MRM chromatograms obtained for selected pesticides at the LOQ are provided in Figure S1 of the Supplementary Material, illustrating chromatographic selectivity and analyte detection under the most demanding validated analytical conditions. Recovery and precision data for all pesticides are presented in Table S2. Overall, satisfactory performance was achieved for the vast majority of analytes. Of the 144 recovery results generated during validation, 131 (91.0%) fulfilled the SANTE recovery criterion of 70–120%, whereas more than 98% of the calculated relative standard deviation (RSD) values complied with the recommended precision criterion of ≤20%. Figure 2 shows the average recovery and RSD values for all pesticides and spiking levels (*n* = 15).

Deviations from the recovery criteria occurred for only a few compounds. Pirimiphos-ethyl consistently exhibited recoveries below the recommended range, whereas metalaxyl, malathion, triflumizole, heptachlor *cis*, DDD, iprodione, and esfenvalerate exceeded the upper recovery limit at a single concentration level. Propiconazole showed recoveries below 70% at two fortification levels, while fenamiphos fell slightly below the acceptance criterion at the highest fortification level. In terms of precision, only heptachlor *trans* exceeded the recommended RSD threshold, and this occurred exclusively at the lowest fortification level. Overall, these observations indicate compound-dependent analytical behavior and suggest that residual matrix-related effects may still influence the performance of certain pesticides despite the application of matrix-matched calibration, APs, and IS correction. Nevertheless, the majority of pesticides demonstrated satisfactory trueness and precision across the evaluated concentration range.

The validation results obtained in this study are comparable to those reported for QuEChERS-based multi-residue methodologies applied to soils. Many reported procedures have focused on a limited number of analytes, typically between 1 and 40 pesticides [29,30,31,32,33,34,35,36], or on specific pesticide classes such as organochlorines [37,38,39], organophosphates [40], pyrethroids [41], or chloroacetanilides [42]. Although some multiresidue methods have reported broader scopes [43,44,45], the proposed methodology enables the simultaneous determination of 48 pesticides belonging to multiple chemical families. In terms of analytical performance, numerous QuEChERS-based methods have reported recoveries within the generally accepted 70–120% range [30,31,34,36,42,43]. Nevertheless, previous studies have reported recovery ranges of 57–124% [38], 54–103% [32], 55–118% [35], and 65–103% [37], indicating that deviations from the ideal recovery interval are not uncommon in multiresidue soil analysis. Similar observations were made in the present study, where 131 of 144 recovery results (91.0%) fulfilled the SANTE acceptance criterion of 70–120%, while deviations were restricted to a limited number of analytes and fortification levels. Furthermore, while recovery performance is routinely reported in previously published methodologies, systematic evaluation of MEs is considerably less common [10,11]. In contrast, the present work included a dedicated assessment of MEs for all 48 target pesticides through comparison of matrix-matched and solvent-based calibration curves. This evaluation showed that 60.4% of analytes exhibited soft MEs, 37.5% exhibited medium MEs, and only one pesticide exhibited a strong ME. The combined use of QuEChERS extraction, Florisil-assisted dSPE clean-up, APs, IS correction, and matrix-matched calibration ensured reliable analytical performance across a wide range of pesticide classes. Overall, the method fulfilled SANTE performance criteria in terms of linearity, trueness, and precision for the vast majority of analytes, while effectively managing MEs. These findings confirm the suitability of the proposed methodology for multi-residue pesticide monitoring in tropical agricultural soils, as represented by the validation matrix characterized by low organic carbon content, low cation exchange capacity, and predominantly sandy texture.

### 2.3. Application of the Validated Method to Agricultural Soil Samples

The validated QuEChERS-GC-MS/MS method was applied to agricultural soil samples collected from five sampling locations within the tropical region of Puerto Carreño (Colombian Orinoquia). All samples corresponded to the same land-use category and exhibited comparable physicochemical characteristics, as previously described (Table 1), ensuring consistency between method performance validation and real sample application.

All collected soil samples were analyzed according to the optimized analytical protocol. Each sample was processed following the same extraction, clean-up, and instrumental conditions used during method validation. Identification criteria were based on retention time matching and specific multiple reaction monitoring (MRM) transitions. No residues of any of the 48 target pesticides were detected above the method reporting threshold in any of the analyzed samples.

The absence of detectable residues should be interpreted within the analytical scope of the method. Although the target list included 48 pesticides representing the most relevant compound classes historically and currently applied in Colombian agricultural practice, particularly organophosphorus, organochlorine, triazine, and pyrethroid pesticides [46], no residues were detected above the reporting threshold in any of the analyzed samples. The occurrence and persistence of pesticide residues in soils may be influenced by multiple environmental and anthropogenic factors, including pesticide application practices, land-use management, degradation processes, climatic conditions, and soil physicochemical properties [47,48,49]. However, the present study did not include agronomic management records or historical pesticide application data. Therefore, the absence of detectable residues cannot be attributed to any specific factor and only indicates that none of the target pesticides were present above the method reporting threshold under the conditions evaluated in this study.

## 3. Materials and Methods

### 3.1. Reagents and Standards

Reagents used for soil physicochemical characterization included sodium hexametaphosphate [(NaPO_3_)_6_], sodium carbonate (Na_2_CO_3_), potassium dichromate (K_2_Cr_2_O_7_), concentrated sulfuric acid (H_2_SO_4_, 95–98%), ammonium ferrous sulfate hexahydrate [(NH_4_)_2_Fe(SO_4_)_2_∙6H_2_O], 1,10-phenanthroline (C_12_H_8_N_2_), ammonium acetate (NH_4_CH_3_COO), and Milli-Q water (resistivity > 18.2 MΩ cm at 25 °C). All these reagents were supplied by Merck KGaA (Darmstadt, Germany) or Sigma-Aldrich (St. Louis, MO, USA) and used without further purification.

For pesticide residue analysis, individual analytical pesticide standards for the 48 target pesticides, the procedural IS atrazine-d5, and the injection quality control (QC) compound triphenyl phosphate (TPP) were obtained from Sigma-Aldrich (St. Louis, MO, USA), all with certified purities ≥ 95.9%. The APs (3-ethoxy-1,2-propanediol (ethylglycerol), L-gulonic acid-γ-lactone (gulonolactone), D-sorbitol, and shikimic acid) were also obtained from Sigma-Aldrich (purity ≥ 95.0%). GC-MS-grade ACN, toluene, formic acid (98–100%), methanol, ethyl acetate, and glacial acetic acid (AcOH; ≥99.8%) were supplied by Merck KGaA (Darmstadt, Germany). Anhydrous magnesium sulfate (MgSO_4_) and sodium acetate (NaOAc) were obtained from Sigma-Aldrich (St. Louis, MO, USA). PSA, C18, and Florisil sorbents were purchased from Supelco (Bellefonte, PA, USA).

Individual pesticide stock solutions were prepared at approximately 1000 mg/L in toluene. Stock solutions of atrazine-d5 and TPP were prepared at concentrations of 750 mg/L and 1050 mg/L, respectively. All stock solutions were stored in amber glass vials at −20 °C until use.

Working standard mixtures were prepared in ACN containing 0.05% (*v*/*v*) formic acid. Spiking solutions were prepared to yield final concentrations of 50, 200, and 400 µg/kg in soil samples, while the procedural IS concentration was fixed at 200 µg/kg. Calibration solutions were prepared in the same solvent to obtain concentration levels of 5, 10, 25, 75, 200, 400, and 600 µg/kg sample equivalents for the target analytes, maintaining the IS at 200 µg/kg.

A mixture of APs was prepared in ACN/water (4:1, *v*/*v*) containing 0.5% (*v*/*v*) formic acid, at concentrations of 100 g/L ethylglycerol, 10 g/L gulonolactone, 10 g/L D-sorbitol, and 5 g/L shikimic acid. This solution was added to the final extracts immediately prior to instrumental analysis.

### 3.2. Study Area and Soil Sampling

The study was conducted in agricultural areas located in the municipality of Puerto Carreño (centered at 6°10′57″ N 67°30′47″ W), Vichada Department, Colombian Orinoquia, in the framework of the international cooperation project entitled “Sustainable ecosystem around renewable energies for Puerto Carreño—Vichada” funded by the French Chancellery. The region is characterized by extensive savanna landscapes and increasing interest in the development of sustainable agricultural practices. The selected area corresponds to zones currently considered for the implementation of organic agroecosystems, where baseline information on soil quality and potential contamination is required to support land-use planning and management strategies.

Soil samples were collected across five sampling locations (0.1 ha each). Within each location, eight individual samples were collected from the nodes of an eight-point square-grid systematic sampling design, following NSW EPA recommendations for contaminated land assessment to prevent chemical dilution and preserve spatial variance [50]. Surface soil samples were collected from the 0–10 cm layer, corresponding to the soil horizon most directly influenced by agricultural management practices and potential pesticide inputs.

For physicochemical characterization, the eight subsamples collected within each sampling location were homogenized to obtain a composite sample representative of that location. In contrast, pesticide residue analyses were performed on each subsample to preserve spatial variability and avoid potential dilution of localized contamination.

Following physicochemical characterization, one sampling location was selected as the validation matrix because its physicochemical properties were representative of the soils investigated in this study. This soil was subsequently used for the preparation of matrix-matched calibration standards and recovery experiments.

Samples were transferred to airtight amber glass containers and transported under dark conditions to minimize exposure to sunlight and temperature fluctuations. Upon arrival at the laboratory, samples were processed according to the procedures described above for physicochemical characterization and pesticide residue analysis. Prior to analysis, soils were air-dried under ambient laboratory conditions, sieved through a 2 mm stainless-steel mesh, and stored in airtight amber glass containers at 4 °C until analysis.

### 3.3. Physicochemical Analysis of Soil Samples

Prior to pesticide residue analysis, selected physicochemical properties of the soil samples were determined to characterize the matrices used for method validation and application. The evaluated parameters included texture, pH, total organic carbon, and cation exchange capacity. The physicochemical characterization was performed to assess the representativeness and homogeneity of the investigated soils for method validation purposes rather than to establish quantitative relationships between soil properties and MEs. Such correlations would require the evaluation of MEs across a broader range of soils exhibiting contrasting physicochemical characteristics.

Soil texture was determined using the modified Bouyoucos hydrometer method [18]. Briefly, soil particles (50 g) were dispersed in an aqueous solution containing a chemical dispersant ((NaPO_3_)_6_ combined with Na_2_CO_3_) and mechanically agitated to ensure complete disaggregation. Particle-size distribution was calculated from hydrometer readings taken after 40 s and 2 h of sedimentation.

Soil pH was measured potentiometrically in a soil-to-water suspension (1:2, *w*/*v*) [51]. Briefly, 20 g of soil was mixed with Milli-Q water, agitated for 1 min, and allowed to stand for 5 min before measuring the pH.

Total organic carbon was determined using the Walkley-Black dichromate oxidation method [52]. In this procedure, 1 g of soil was treated with K_2_Cr_2_O_7_ and H_2_SO_4_, followed by titration of the excess oxidant with (NH_4_)_2_Fe(SO_4_)_2_∙6H_2_O using C_12_H_8_N_2_ as an indicator. Results were calculated from the consumption of (NH_4_)_2_Fe(SO_4_)_2_∙6H_2_O relative to a procedural blank.

Cation exchange capacity was determined using the ammonium acetate method [53]. Briefly, 5 g of soil was saturated with NH_4_CH_3_COO, then washed sequentially, and exchangeable ammonium was displaced with NaCl. The released ammonium was quantified by titration with standardized NaOH after treatment with formaldehyde.

### 3.4. Pesticide Residue Analysis

#### 3.4.1. QuEChERS Extraction Procedure

**Figure 3:**
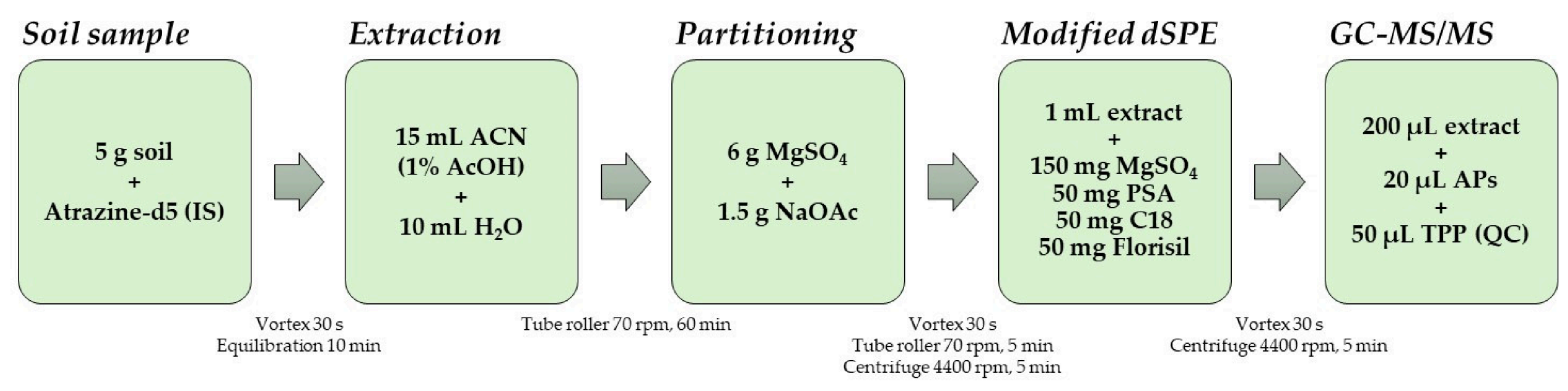
Schematic representation of the modified QuEChERS extraction and dSPE clean-up procedure employed for the determination of 48 multi-class pesticide residues in tropical agricultural soils prior to GC-MS/MS analysis.

Pesticide residues were extracted from soil samples using a modified QuEChERS method based on the official AOAC 2007.01 procedure [54], adapted for tropical agricultural soil matrices. The sample preparation workflow consisted of two main stages: extraction and dSPE clean-up. A schematic overview of the procedure is presented in Figure 3.

Briefly, 5 g of homogenized soil was weighed into a 50 mL polypropylene centrifuge tube, fortified with atrazine-d5 as the procedural IS, vortexed for 30 s, and allowed to equilibrate for 10 min. Subsequently, 15 mL of ACN containing 1% (*v*/*v*) AcOH and 10 mL of Milli-Q water were added. The mixture was agitated using a tube roller (MX-T6-Pro, Scilogex, Rocky Hill, CT, USA) at 70 rpm for 60 min. Following extraction, 6 g of anhydrous MgSO_4_ and 1.5 g of NaOAc were added to promote phase separation. The tubes were vortex-mixed for 30 s, agitated on the tube roller for 5 min at 70 rpm, and centrifuged at 4400 rpm for 5 min. Clean-up of the organic extract was performed by transferring 1 mL of the supernatant into a 2 mL centrifuge tube containing 150 mg of anhydrous MgSO_4_, 50 mg of PSA, 50 mg of C18, and 50 mg of Florisil. The mixture was vortex-mixed for 30 s and centrifuged at 4400 rpm for 5 min. An aliquot of 200 µL of the cleaned extract was transferred to an autosampler vial and mixed with 20 µL of the AP solution together with 50 µL of TPP solution. Finally, 1 µL of the resulting extract was injected into the GC-MS/MS system for analysis.

#### 3.4.2. GC-MS/MS Conditions

Instrumental analyses were performed using a GCMS-TQ8040 gas chromatograph coupled to a triple quadrupole mass spectrometer (Shimadzu, Kyoto, Japan) equipped with an electron ionization source and an AOC-20i/s autosampler. Data acquisition and processing were conducted using GC-MS solution software (Shimadzu, Kyoto, Japan). Chromatographic separation was achieved on an SH-Rxi-5Sil MS capillary column (30 m, 0.25 mm i.d., 0.25 μm film thickness; Shimadzu, Kyoto, Japan). Helium (purity ≥ 99.999%) was used as the carrier gas at a constant flow rate of 1.2 mL/min. The oven temperature program was as follows: initial temperature of 50 °C held for 1 min, ramped to 180 °C at 25 °C/min, then to 230 °C at 5 °C/min, and finally to 290 °C at 25 °C/min, where it was held for 6 min. The total chromatographic run time was 24.6 min. This temperature gradient was selected to achieve adequate separation of pesticides belonging to different chemical classes within a reasonable analysis time. The final program provided satisfactory resolution of the target compounds while minimizing peak overlap and maintaining appropriate peak shapes throughout the chromatographic run. Sample injections (1 μL) were performed in splitless mode at an injector temperature of 250 °C, incorporating methanol, ethyl acetate, and ACN wash steps between injections. The transfer line and ion source temperatures were maintained at 250 °C and 300 °C, respectively. Prior to each analytical sequence, an automatic mass spectrometer tuning procedure was performed to verify instrument performance. Detection was carried out in MRM mode. Quantification was based on the most intense transition (quantifier ion), whereas confirmation was performed using two additional qualifier transitions. Collision energies were individually optimized for each compound within the range of 5–40 eV. Table 2 summarizes the retention times, quantifier and qualifier transitions, and optimized collision energies for the 48 target pesticides, the procedural IS (atrazine-d5), and the injection QC compound (TPP).

#### 3.4.3. Validation Studies

The developed QuEChERS-GC-MS/MS procedure was validated in terms of linearity, MEs, recovery, and precision, according to the performance criteria established in the SANTE/2020/12830 (Rev. 2) guidance document for pesticide residue analysis in soil [23]. Method selectivity was evaluated through retention-time and MRM-transition criteria. Compound identification was based on agreement of retention times with those of matrix-matched calibration standards and on the relative abundance of characteristic MRM transitions. Retention-time deviations did not exceed ±0.1 min relative to the corresponding calibration standards, while deviations of qualifier-to-quantifier ion ratios remained below 30%, in accordance with the SANTE/2020/12830 (Rev. 2) guidance document.

Linearity was assessed using matrix-matched calibration curves prepared from blank soil extracts. Calibration standards and atrazine-d5 were added to the cleaned blank extracts after the QuEChERS extraction and clean-up procedure to obtain final concentrations of 5, 10, 25, 75, 200, 400, and 600 µg/kg for the target pesticides and 200 µg/kg for the IS. Calibration curves were constructed by plotting analyte-to-internal-standard peak area ratios against concentration.

To evaluate MEs, solvent-based calibration curves were prepared at the same concentration levels and under identical instrumental conditions as the matrix-matched standards. For this purpose, 200 µL of ACN was used instead of the cleaned soil extract while maintaining the same concentrations of analytes, IS, APs, and QC standard. MEs were calculated according to Equation (1):

(1) $$ME\left(\%\right)=\left[\left(\frac{S{lope}_{matrix}}{Slo{pe}_{solvent}}\right)-1\right]\times 100,$$

where $S{lope}_{matrix}$ and $Slo{pe}_{solvent}$ correspond to the slopes of the matrix-matched and solvent-based calibration curves, respectively. Negative values indicate signal suppression, whereas positive values indicate signal enhancement [26].

Method accuracy and precision were evaluated through recovery experiments performed at three fortification levels (50, 200, and 400 µg/kg). According to the SANTE/2020/12830 (Rev. 2) guidance document, the LOQ corresponds to the lowest validated concentration level demonstrating acceptable recovery and precision. Therefore, 50 µg/kg (0.05 mg/kg), corresponding to the lowest fortification level included in the validation study, was selected as the method LOQ. This value is also consistent with the concentration generally recommended by SANTE for pesticide residue determination in soil. Concentrations below the LOQ were included only for calibration purposes to evaluate linearity and instrumental response and were not considered part of the validated concentration range. Blank soil samples were fortified with the appropriate pesticide standard mixture and atrazine-d5 (200 µg/kg) and allowed to equilibrate at room temperature for 60 min prior to extraction to facilitate analyte sorption onto the soil matrix and thereby provide recovery estimates under conditions more representative of naturally contaminated samples. Five replicate samples were prepared and analyzed at each fortification level (*n* = 5). Recoveries were calculated as the percentage ratio between measured and fortified concentrations, whereas method precision was expressed as RSD (%). Quantification was performed using the corresponding matrix-matched calibration curves.

## 4. Conclusions

This study presents a validated QuEChERS-GC-MS/MS method for the multi-residue determination of 48 pesticides, covering major chemical classes commonly applied in agriculture, in tropical soils from the Colombian Orinoquia. The method, developed under SANTE/2020/12830 (Rev. 2) guidelines, demonstrated adequate analytical performance in terms of linearity, trueness, and precision for trace-level pesticide screening and quantification.

A key contribution of this work is the adaptation of the QuEChERS approach to tropical agricultural soils. The incorporation of a modified dSPE clean-up based on a combination of PSA, C18, and Florisil, together with APs, a deuterated IS, and matrix-matched calibration, proved effective for controlling MEs during GC-MS/MS analysis. Most compounds exhibited soft to moderate MEs, indicating that the combined purification and signal-compensation strategy provided reliable performance across a broad range of pesticide classes.

Recovery experiments further confirmed the reliability of the method, with the majority of analytes fulfilling the performance criteria established by SANTE guidelines. These results demonstrate the suitability of the proposed analytical workflow for pesticide residue determination in tropical soil matrices that remain underrepresented in the current literature.

Application of the validated method to soil samples collected from five agricultural locations revealed no detectable residues of the target pesticides above the method reporting threshold. This result demonstrates the applicability of the proposed method for pesticide residue assessment in tropical agricultural soils.

Overall, the proposed method provides a robust, selective, and reliable analytical tool for routine monitoring of pesticide residues in tropical agricultural soils with similar characteristics, and may be applied in future studies aimed at evaluating environmental contamination and supporting sustainable land-use management strategies in similar agroecosystems.

## Figures and Tables

**Table 1:** Physicochemical characteristics of the studied soils.

Parameter	Site 1 ^1^	Site 2	Site 3	Site 4	Site 5
Texture	Sandy	Sandy loam	Sandy	Sandy	Sandy
pH	5.4	5.2	5.7	5.6	5.3
Total organic carbon (%)	0.4	0.6	0.1	0.8	0.5
Cation exchange capacity (meq/100 g)	6.8	7.0	5.6	8.1	5.6

^1^ Selected as a validation matrix for matrix-matched calibration and recovery experiments.

**Table 2:** Retention times, quantifier and qualifier transitions, and collision energies in GC-MS/MS analyses of the target pesticides, atrazine-d5, and TPP.

Analyte	Retention Time (min)	Quantifier Transition (*m*/*z*)	Collision Energy (V)	Qualifier Transition (*m*/*z*)	Collision Energy (V)	Qualifier Transition (*m*/*z*)	Collision Energy (V)
Propoxur	5.66	110.0 → 64.0	20	152.0 → 110.0	10	110.0 → 82.0	15
Carbofuran	6.44	164.0 → 149.0	10	149.0 → 103.0	15	149.0 → 131.0	15
Diphenylamine	8.91	168.0 → 167.0	10	169.0 → 168.0	5	169.0 → 167.0	5
Chlorpropham	9.11	213.0 → 171.0	5	213.0 → 127.0	15	127.0 → 90.0	15
Dicloran	9.95	206.0 → 176.0	10	124.0 → 97.0	10	176.0 → 124.0	10
Atrazine-d5 (IS)	10.05	205.0 → 127.0	10	220.0 → 178.0	5	205.0 → 178.0	5
Atrazine	10.08	215.0 → 173.0	5	215.0 → 200.0	10	200.0 → 173.0	10
Lindane	10.39	183.0 → 181.0	5	219.0 → 181.0	10	181.0 → 109.0	25
Propyzamide	10.08	175.0 → 173.0	15	175.0 → 173.0	5	173.0 → 109.0	10
β-HCH	10.39	183.0 → 147.0	15	183.0 → 145.0	20	183.0 → 111.0	35
α-HCH	10.39	181.0 → 145.0	15	219.0 → 181.0	5	181.0 → 74.0	35
Chlorpyrifos-methyl	11.72	288.0 → 286.0	5	286.0 → 93.0	25	286.0 → 125.0	25
Parathion-methyl	11.87	263.0 → 109.0	15	109.0 → 79.0	10	263.0 → 125.0	10
Alachlor	11.90	188.0 → 160.0	10	160.0 → 132.0	15	188.0 → 146.0	10
Tolclofos-methyl	11.91	265.0 → 250.0	15	267.0 → 265.0	5	265.0 → 125.0	5
Metalaxyl	12.07	206.0 → 132.0	20	160.0 → 146.0	10	206.0 → 160.0	15
Pirimiphos-methyl	12.43	290.0 → 233.0	10	305.0 → 290.0	10	290.0 → 276.0	10
Fenitrothion	12.53	277.0 → 260.0	5	277.0 → 109.0	20	125.0 → 109.0	15
Malathion	12.73	127.0 → 99.0	5	173.0 → 127.0	5	125.0 → 93.0	5
Chlorpyrifos	12.98	314.0 → 258.0	15	199.0 → 171.0	15	197.0 → 97.0	15
Fenthion	13.08	278.0 → 109.0	20	278.0 → 169.0	20	278.0 → 245.0	10
Triadimefon	13.29	208.0 → 181.0	10	208.0 → 128.0	15	128.0 → 85.0	10
Aldrin	13.17	263.0 → 193.0	25	263.0 → 191.0	30	263.0 → 228.0	25
Pirimiphos-ethyl	13.57	333.0 → 168.0	25	333.0 → 318.0	10	318.0 → 180.0	10
Heptachlor	14.38	274.0 → 239.0	15	274.0 → 141.0	30	100.0 → 65.0	5
Penconazole	14.13	161.0 → 159.0	5	248.0 → 159.0	25	248.0 → 161.0	5
Triflumizole	14.25	278.0 → 206.0	5	278.0 → 206.0	20	278.0 → 179.0	5
Methidathion	14.86	145.0 → 85.0	10	145.0 → 58.0	15	145.0 → 93.0	10
Heptachlor *cis*	14.39	253.0 → 219.0	35	253.0 → 219.0	15	253.0 → 205.0	20
Heptachlor *trans*	14.39	183.0 → 147.0	25	353.0 → 220.0	35	183.0 → 147.0	20
Fenamiphos	15.52	303.0 → 288.0	10	303.0 → 154.0	20	303.0 → 217.0	10
Endosulfan A	15.39	241.0 → 206.0	20	241.0 → 204.0	15	206.0 → 170.0	25
Myclobutanil	16.15	179.0 → 125.0	15	179.0 → 150.0	10	179.0 → 82.0	10
Dieldrin	16.24	263.0 → 191.0	30	263.0 → 193.0	30	263.0 → 81.0	35
Oxyfluorfen	16.21	252.0 → 223.0	15	252.0 → 223.0	15	300.0 → 252.0	15
Buprofezin	16.27	172.0 → 57.0	20	175.0 → 172.0	5	105.0 → 83.0	10
Endrin	16.24	263.0 → 228.0	5	263.0 → 81.0	35	263.0 → 228.0	20
Ethion	16.15	153.0 → 125.0	10	153.0 → 125.0	5	231.0 → 153.0	5
DDE	16.05	246.0 → 176.0	25	210.0 → 176.0	35	246.0 → 210.0	35
DDD	15.38	235.0 → 200.0	5	235.0 → 200.0	30	165.0 → 147.0	15
Propiconazole	17.86	175.0 → 173.0	5	259.0 → 69.0	10	259.0 → 173.0	10
Tebuconazole	18.13	250.0 → 125.0	20	250.0 → 70.0	10	125.0 → 70.0	10
TPP (QC)	18.19	326.0 → 325.0	5	326.0 → 169.0	25	326.0 → 215.0	25
Iprodione	18.52	314.0 → 56.0	25	314.0 → 187.0	5	314.0 → 245.0	5
EPN	18.66	185.0 → 157.0	5	169.0 → 157.0	5	157.0 → 141.0	10
Pyriproxyfen	19.31	136.0 → 78.0	20	136.0 → 96.0	15	136.0 → 78.0	15
DDT	17.16	235.0 → 165.0	15	200.0 → 165.0	5	235.0 → 163.0	20
Fenvalerate	22.21	167.0 → 125.0	10	181.0 → 152.0	25	225.0 → 125.0	40
Esfenvalerate	22.51	167.0 → 125.0	10	225.0 → 167.0	20	225.0 → 181.0	20
Deltamethrin	23.05	253.0 → 172.0	10	253.0 → 174.0	10	253.0 → 181.0	10

## Data Availability

Dataset available on request from the authors.

## Supplementary Materials

The following supporting information can be downloaded at: https://www.mdpi.com/article/10.3390/molecules31152564/s1, Table S1: Matrix-matched and solvent calibration data of the selected pesticides and matrix effect percentages; Figure S1: Representative MRM chromatograms for five pesticides covering different retention-time regions, obtained from soil samples fortified at the method LOQ (50 µg/kg): (A) propoxur, (B) β-HCH, (C) lindane, (D) aldrin, and (E) DDT. The selected compounds include β-HCH and lindane, which exhibit similar retention times but were successfully distinguished through characteristic MRM transitions. The chromatograms illustrate chromatographic selectivity and analyte detection at the lowest validated concentration level of the method, corresponding to the most demanding validated analytical conditions; Table S2: Average recoveries (RSDs) of the selected pesticides in the validation sample (*n* = 5 at each spiking level).

## Abbreviations

The following abbreviations are used in this manuscript:

ACN	Acetonitrile
AP	Analyte protectant
C18	Octadecylsilane
dSPE	Dispersive solid-phase extraction
GC-MS/MS	Gas chromatography-tandem mass spectrometry
IS	Internal standard
ME	Matrix effect
MRM	Multiple reaction monitoring
PSA	Primary secondary amine
QC	Quality control
QuEChERS	Quick, Easy, Cheap, Effective, Rugged, and Safe
R^2^	Coefficient of determination
RSD	Relative standard deviation
TPP	Triphenyl phosphate
