# Supplementary material for: QuEChERS-GC-MS/MS Analysis of Multi-Class Pesticide Residues in Tropical Agricultural Soils
Source: Molecules. 2026 Jul 23;31(15):2564. doi: 10.3390/molecules31152564 (PMC13468114; doi:10.3390/molecules31152564)
Supplement: Supplementary file 1 [file molecules-31-02564-s001.zip › molecules-4438152-supplementary.pdf]

Supplementary material

QuEChERS-GC-MS/MS Analysis of Multi-Class Pesticide Residues in Tropical Agricultural Soils

Diego Alejandro Riaño-Herrera[1], Laura Herrera-Paiva[1], Julien Gwendal Chenet[1,†], Alberto Uribe-Jongbloed[1], Diana Angélica Varela-Martínez[2], Miguel Ángel González-Curbelo[2,*]

[1] Departamento de Ingeniería Ambiental y Energías, Facultad de Ingeniería, Universidad EAN. Calle 79 nº 11-45, Bogotá D.C., Colombia. dariano@universidadean.edu.co (D.A.R.-H.); lherrera1451@universidadean.edu.co (L.H.-P.); auribejo@universidadean.edu.co (A.U-J.)

[2] Departamento de Ciencias Básicas, Facultad de Ingeniería, Universidad EAN. Calle 79 nº 11-45, Bogotá D.C., Colombia. davarela@universidadean.edu.co (D.A.V.-M.)

† Deceased

* Correspondence: magonzalez@universidadean.edu.co

Table S1. Matrix-matched and solvent calibration data of the selected pesticides and matrix effect percentages.

Pesticide	Sample	Studied linear range (µg/kg)	Regression equation (n=7)		$s_{y/x}$	R^2	ME (%)
			$b \pm s_b \cdot t_{(0.05;7)}$	$a \pm s_a \cdot t_{(0.05;7)}$			
Propoxur	Soil	5-600	$1.21 \cdot 10^{-2} \pm 4.50 \cdot 10^{-4}$	$1.61 \cdot 10^{+2} \pm 3.47 \cdot 10^{+1}$	$2.66 \cdot 10^{+1}$	0.9993	-29.7
	Solvent calibration	5-600	$1.72 \cdot 10^{-2} \pm 2.35 \cdot 10^{-3}$	$3.11 \cdot 10^{-1} \pm 6.73 \cdot 10^{-1}$	$4.85 \cdot 10^{-1}$	0.9959	-
Carbofuran	Soil	5-600	$5.26 \cdot 10^{-3} \pm 2.41 \cdot 10^{-4}$	$-1.99 \cdot 10^{-3} \pm 6.89 \cdot 10^{-2}$	$5.27 \cdot 10^{-2}$	0.9982	12.4
	Solvent calibration	5-600	$4.68 \cdot 10^{-3} \pm 2.13 \cdot 10^{-4}$	$-5.07 \cdot 10^{-3} \pm 6.10 \cdot 10^{-2}$	$4.39 \cdot 10^{-2}$	0.9984	-
Diphenylamine	Soil	5-600	$1.41 \cdot 10^{-1} \pm 5.17 \cdot 10^{-3}$	$-2.26 \cdot 10^{-1} \pm 1.48 \cdot 10$	$1.13 \cdot 10$	0.9988	11.9
	Solvent calibration	5-600	$1.26 \cdot 10^{-1} \pm 9.10 \cdot 10^{-3}$	$-7.65 \cdot 10^{-1} \pm 2.60 \cdot 10$	$1.88 \cdot 10$	0.9960	-
Chlorpropham	Soil	5-600	$6.89 \cdot 10^{-3} \pm 1.09 \cdot 10^{-4}$	$-1.95 \cdot 10^{-2} \pm 3.12 \cdot 10^{-2}$	$2.39 \cdot 10^{-2}$	0.9998	6.2
	Solvent calibration	5-600	$6.49 \cdot 10^{-3} \pm 4.06 \cdot 10^{-4}$	$-3.59 \cdot 10^{-2} \pm 1.16 \cdot 10^{-1}$	$8.36 \cdot 10^{-2}$	0.9970	-
Dicloran	Soil	5-600	$4.93 \cdot 10^{-3} \pm 9.54 \cdot 10^{-5}$	$-2.34 \cdot 10^{-2} \pm 2.73 \cdot 10^{-2}$	$2.09 \cdot 10^{-2}$	0.9997	-9.2
	Solvent calibration	5-600	$5.43 \cdot 10^{-3} \pm 4.34 \cdot 10^{-4}$	$-6.10 \cdot 10^{-2} \pm 1.24 \cdot 10^{-1}$	$8.94 \cdot 10^{-2}$	0.9951	-
Atrazine	Soil	5-600	$5.18 \cdot 10^{-3} \pm 8.76 \cdot 10^{-5}$	$-4.84 \cdot 10^{-3} \pm 2.51 \cdot 10^{-2}$	$1.92 \cdot 10^{-2}$	0.9998	-1.0
	Solvent calibration	5-600	$5.23 \cdot 10^{-3} \pm 1.69 \cdot 10^{-4}$	$-3.33 \cdot 10^{-2} \pm 4.85 \cdot 10^{-2}$	$3.49 \cdot 10^{-2}$	0.9992	-
Lindane	Soil	5-600	$1.70 \cdot 10^{-2} \pm 2.94 \cdot 10^{-4}$	$-3.57 \cdot 10^{-3} \pm 8.41 \cdot 10^{-2}$	$6.43 \cdot 10^{-2}$	0.9997	-21.7
	Solvent calibration	5-600	$2.17 \cdot 10^{-2} \pm 1.19 \cdot 10^{-3}$	$-8.47 \cdot 10^{-2} \pm 3.40 \cdot 10^{-1}$	$2.45 \cdot 10^{-1}$	0.9977	-
Propyzamide	Soil	5-600	$3.30 \cdot 10^{-3} \pm 1.55 \cdot 10^{-4}$	$-2.91 \cdot 10^{-3} \pm 4.43 \cdot 10^{-2}$	$3.38 \cdot 10^{-2}$	0.9981	-5.7
	Solvent calibration	5-600	$3.50 \cdot 10^{-3} \pm 2.52 \cdot 10^{-4}$	$-2.37 \cdot 10^{-3} \pm 7.22 \cdot 10^{-2}$	$5.20 \cdot 10^{-2}$	0.9960	-
β-HCH	Soil	5-600	$2.26 \cdot 10^{-2} \pm 2.36 \cdot 10^{-4}$	$-5.11 \cdot 10^{-2} \pm 6.76 \cdot 10^{-2}$	$5.17 \cdot 10^{-2}$	0.9999	-17.2
	Solvent calibration	5-600	$2.73 \cdot 10^{-2} \pm 1.16 \cdot 10^{-3}$	$-9.57 \cdot 10^{-2} \pm 3.33 \cdot 10^{-1}$	$2.40 \cdot 10^{-1}$	0.9986	-
α-HCH	Soil	5-600	$3.21 \cdot 10^{-2} \pm 2.55 \cdot 10^{-4}$	$-5.56 \cdot 10^{-2} \pm 7.29 \cdot 10^{-2}$	$5.57 \cdot 10^{-2}$	0.9999	-17.5
	Solvent calibration	5-600	$3.89 \cdot 10^{-2} \pm 1.79 \cdot 10^{-3}$	$-1.35 \cdot 10^{-1} \pm 5.11 \cdot 10^{-1}$	$3.68 \cdot 10^{-1}$	0.9984	-
Chlorpyrifos-methyl	Soil	5-600	$1.62 \cdot 10^{-2} \pm 2.28 \cdot 10^{-4}$	$1.15 \cdot 10^{-2} \pm 6.52 \cdot 10^{-2}$	$4.99 \cdot 10^{-2}$	0.9998	-16.1
	Solvent calibration	5-600	$1.93 \cdot 10^{-2} \pm 1.19 \cdot 10^{-3}$	$-1.23 \cdot 10^{-1} \pm 3.41 \cdot 10^{-1}$	$2.46 \cdot 10^{-1}$	0.9971	-
Parathion-methyl	Soil	5-600	$5.45 \cdot 10^{-3} \pm 1.46 \cdot 10^{-4}$	$-2.40 \cdot 10^{-2} \pm 4.18 \cdot 10^{-2}$	$3.20 \cdot 10^{-2}$	0.9994	-19.9
	Solvent calibration	5-600	$6.80 \cdot 10^{-3} \pm 4.74 \cdot 10^{-4}$	$-7.73 \cdot 10^{-2} \pm 1.36 \cdot 10^{-1}$	$9.76 \cdot 10^{-2}$	0.9963	-
Alachlor	Soil	5-600	$1.48 \cdot 10^{-2} \pm 2.33 \cdot 10^{-4}$	$-4.96 \cdot 10^{-3} \pm 6.66 \cdot 10^{-2}$	$5.10 \cdot 10^{-2}$	0.9998	-12.9

	Solvent calibration	5-600	$1.70\cdot10^{-2} \pm 8.63\cdot10^{-4}$	$-6.34\cdot10^{-2} \pm 2.47\cdot10^{-1}$	$1.78\cdot10^{-1}$	0.9980	-
Tolclofos-methyl	Soil	5-600	$1.95\cdot10^{-2} \pm 1.68\cdot10^{-4}$	$7.35\cdot10^{-3} \pm 4.80\cdot10^{-2}$	$3.67\cdot10^{-2}$	0.9999	-9.7
	Solvent calibration	5-600	$2.16\cdot10^{-2} \pm 9.90\cdot10^{-4}$	$-9.16\cdot10^{-2} \pm 2.83\cdot10^{-1}$	$2.04\cdot10^{-1}$	0.9984	-
Metalaxyl	Soil	5-600	$5.86\cdot10^{-3} \pm 1.97\cdot10^{-4}$	$3.32\cdot10^{-4} \pm 5.63\cdot10^{-2}$	$4.30\cdot10^{-2}$	0.9990	-12.3
	Solvent calibration	5-600	$6.68\cdot10^{-3} \pm 2.89\cdot10^{-4}$	$-4.06\cdot10^{-2} \pm 8.26\cdot10^{-2}$	$5.95\cdot10^{-2}$	0.9986	-
Pirimiphos-methyl	Soil	5-600	$7.42\cdot10^{-3} \pm 8.58\cdot10^{-5}$	$-2.18\cdot10^{-2} \pm 2.46\cdot10^{-2}$	$1.88\cdot10^{-2}$	0.9999	-6.8
	Solvent calibration	5-600	$7.96\cdot10^{-3} \pm 4.79\cdot10^{-4}$	$-6.77\cdot10^{-2} \pm 1.37\cdot10^{-1}$	$9.88\cdot10^{-2}$	0.9972	-
Fenitrothion	Soil	5-600	$6.50\cdot10^{-3} \pm 1.48\cdot10^{-4}$	$-5.02\cdot10^{-2} \pm 4.24\cdot10^{-2}$	$3.25\cdot10^{-2}$	0.9995	-15.7
	Solvent calibration	5-600	$7.71\cdot10^{-3} \pm 7.02\cdot10^{-4}$	$-1.06\cdot10^{-1} \pm 2.01\cdot10^{-1}$	$1.45\cdot10^{-1}$	0.9937	-
Malathion	Soil	5-600	$1.69\cdot10^{-2} \pm 2.54\cdot10^{-4}$	$-2.97\cdot10^{-2} \pm 7.28\cdot10^{-2}$	$5.57\cdot10^{-2}$	0.9998	7.0
	Solvent calibration	5-600	$1.58\cdot10^{-2} \pm 7.45\cdot10^{-4}$	$-8.76\cdot10^{-2} \pm 2.13\cdot10^{-1}$	$1.53\cdot10^{-1}$	0.9983	-
Chlorpyrifos	Soil	5-600	$1.14\cdot10^{-2} \pm 2.29\cdot10^{-4}$	$7.10\cdot10^{-4} \pm 6.57\cdot10^{-2}$	$5.02\cdot10^{-2}$	0.9996	-12.3
	Solvent calibration	5-600	$1.30\cdot10^{-2} \pm 5.48\cdot10^{-4}$	$-7.41\cdot10^{-2} \pm 1.57\cdot10^{-1}$	$1.13\cdot10^{-1}$	0.9986	-
Fenthion	Soil	5-600	$1.84\cdot10^{-2} \pm 1.16\cdot10^{-4}$	$-4.97\cdot10^{-2} \pm 3.31\cdot10^{-2}$	$2.54\cdot10^{-2}$	0.9999	-10.2
	Solvent calibration	5-600	$2.05\cdot10^{-2} \pm 9.70\cdot10^{-4}$	$-1.27\cdot10^{-1} \pm 2.78\cdot10^{-1}$	$2.00\cdot10^{-1}$	0.9983	-
Triadimefon	Soil	5-600	$1.17\cdot10^{-2} \pm 1.70\cdot10^{-4}$	$-1.55\cdot10^{-2} \pm 4.85\cdot10^{-2}$	$3.71\cdot10^{-2}$	0.9998	-15.2
	Solvent calibration	5-600	$1.38\cdot10^{-2} \pm 5.05\cdot10^{-4}$	$-5.71\cdot10^{-2} \pm 1.45\cdot10^{-1}$	$1.04\cdot10^{-1}$	0.9990	-
Aldrin	Soil	5-600	$5.96\cdot10^{-3} \pm 2.56\cdot10^{-5}$	$-6.56\cdot10^{-3} \pm 7.33\cdot10^{-3}$	$5.60\cdot10^{-3}$	0.9999	-15.9
	Solvent calibration	5-600	$7.09\cdot10^{-3} \pm 1.85\cdot10^{-4}$	$-1.01\cdot10^{-2} \pm 5.29\cdot10^{-2}$	$3.81\cdot10^{-2}$	0.9995	-
Pirimiphos-ethyl	Soil	5-600	$2.92\cdot10^{-3} \pm 1.23\cdot10^{-4}$	$-9.09\cdot10^{-3} \pm 3.51\cdot10^{-2}$	$2.69\cdot10^{-2}$	0.9985	-13.9
	Solvent calibration	5-600	$3.39\cdot10^{-3} \pm 1.46\cdot10^{-4}$	$-2.62\cdot10^{-2} \pm 4.18\cdot10^{-2}$	$3.01\cdot10^{-2}$	0.9986	-
Heptachlor	Soil	5-600	$2.08\cdot10^{-3} \pm 2.92\cdot10^{-5}$	$-1.17\cdot10^{-2} \pm 8.36\cdot10^{-3}$	$6.40\cdot10^{-3}$	0.9998	-25.7
	Solvent calibration	5-600	$2.80\cdot10^{-3} \pm 6.43\cdot10^{-5}$	$-2.02\cdot10^{-2} \pm 1.84\cdot10^{-2}$	$1.33\cdot10^{-2}$	0.9996	-
Penconazole	Soil	5-600	$1.91\cdot10^{-2} \pm 2.42\cdot10^{-4}$	$7.68\cdot10^{-2} \pm 6.92\cdot10^{-2}$	$5.29\cdot10^{-2}$	0.9999	-20.4
	Solvent calibration	5-600	$2.40\cdot10^{-2} \pm 1.04\cdot10^{-3}$	$-1.22\cdot10^{-1} \pm 2.97\cdot10^{-1}$	$2.14\cdot10^{-1}$	0.9986	-
Triflumizole	Soil	5-600	$5.56\cdot10^{-5} \pm 8.86\cdot10^{-6}$	$1.65\cdot10^{-3} \pm 2.54\cdot10^{-3}$	$1.94\cdot10^{-3}$	0.9784	-26.5
	Solvent calibration	5-600	$7.56\cdot10^{-5} \pm 1.47\cdot10^{-5}$	$1.77\cdot10^{-3} \pm 4.21\cdot10^{-3}$	$3.03\cdot10^{-3}$	0.9717	-
Methidathion	Soil	5-600	$2.27\cdot10^{-2} \pm 3.71\cdot10^{-4}$	$-6.62\cdot10^{-2} \pm 1.06\cdot10^{-1}$	$8.13\cdot10^{-2}$	0.9998	-29.3

	Solvent calibration	5-600	$3.21\cdot10^{-2} \pm 1.75\cdot10^{-3}$	$-2.83\cdot10^{-1} \pm 5.00\cdot10^{-1}$	$3.60\cdot10^{-1}$	0.9977	-
Heptachlor *cis*	Soil	5-600	$1.94\cdot10^{-3} \pm 4.51\cdot10^{-5}$	$-1.15\cdot10^{-2} \pm 1.29\cdot10^{-2}$	$9.87\cdot10^{-3}$	0.9995	-24.8
	Solvent calibration	5-600	$2.58\cdot10^{-3} \pm 5.80\cdot10^{-5}$	$-1.92\cdot10^{-2} \pm 1.66\cdot10^{-2}$	$1.20\cdot10^{-2}$	0.9996	-
Heptachlor *trans*	Soil	5-600	$1.36\cdot10^{-3} \pm 3.78\cdot10^{-5}$	$-9.37\cdot10^{-3} \pm 1.08\cdot10^{-2}$	$8.27\cdot10^{-3}$	0.9993	-26.1
	Solvent calibration	5-600	$1.84\cdot10^{-3} \pm 4.95\cdot10^{-5}$	$-1.31\cdot10^{-2} \pm 1.42\cdot10^{-2}$	$1.02\cdot10^{-2}$	0.9994	-
Fenamiphos	Soil	5-600	$3.09\cdot10^{-3} \pm 5.98\cdot10^{-5}$	$-2.12\cdot10^{-2} \pm 1.71\cdot10^{-2}$	$1.31\cdot10^{-2}$	0.9997	41.1
	Solvent calibration	5-600	$2.19\cdot10^{-3} \pm 2.87\cdot10^{-4}$	$-4.33\cdot10^{-2} \pm 8.20\cdot10^{-2}$	$5.91\cdot10^{-2}$	0.9871	-
Endosulfan A	Soil	5-600	$4.77\cdot10^{-3} \pm 2.77\cdot10^{-5}$	$-1.10\cdot10^{-2} \pm 7.91\cdot10^{-3}$	$6.05\cdot10^{-3}$	0.9999	-26.7
	Solvent calibration	5-600	$6.51\cdot10^{-3} \pm 1.98\cdot10^{-4}$	$-2.51\cdot10^{-2} \pm 5.68\cdot10^{-2}$	$4.09\cdot10^{-2}$	0.9993	-
Myclobutanil	Soil	5-600	$4.62\cdot10^{-2} \pm 3.89\cdot10^{-4}$	$4.63\cdot10^{-2} \pm 1.11\cdot10^{-1}$	$8.51\cdot10^{-2}$	0.9999	-5.1
	Solvent calibration	5-600	$4.87\cdot10^{-2} \pm 2.42\cdot10^{-3}$	$-2.54\cdot10^{-1} \pm 6.92\cdot10^{-1}$	$4.98\cdot10^{-1}$	0.9981	-
Dieldrin	Soil	5-600	$2.86\cdot10^{-3} \pm 4.47\cdot10^{-5}$	$-4.56\cdot10^{-3} \pm 1.28\cdot10^{-2}$	$9.78\cdot10^{-3}$	0.9998	-28.7
	Solvent calibration	5-600	$4.01\cdot10^{-3} \pm 1.56\cdot10^{-4}$	$-2.22\cdot10^{-2} \pm 4.48\cdot10^{-2}$	$3.22\cdot10^{-2}$	0.9988	-
Oxyfluorfen	Soil	5-600	$1.73\cdot10^{-3} \pm 5.98\cdot10^{-5}$	$-6.05\cdot10^{-3} \pm 1.71\cdot10^{-2}$	$1.31\cdot10^{-2}$	0.9990	-19.5
	Solvent calibration	5-600	$2.15\cdot10^{-3} \pm 9.69\cdot10^{-5}$	$-1.97\cdot10^{-2} \pm 2.77\cdot10^{-2}$	$2.00\cdot10^{-2}$	0.9984	-
Buprofezin	Soil	5-600	$7.01\cdot10^{-3} \pm 7.77\cdot10^{-5}$	$-1.14\cdot10^{-2} \pm 2.22\cdot10^{-2}$	$1.70\cdot10^{-2}$	0.9999	-13.9
	Solvent calibration	5-600	$8.14\cdot10^{-3} \pm 4.40\cdot10^{-4}$	$-6.37\cdot10^{-2} \pm 1.26\cdot10^{-1}$	$9.07\cdot10^{-2}$	0.9978	-
Endrin	Soil	5-600	$1.77\cdot10^{-3} \pm 3.97\cdot10^{-5}$	$-8.18\cdot10^{-3} \pm 1.13\cdot10^{-2}$	$8.68\cdot10^{-3}$	0.9996	-27.2
	Solvent calibration	5-600	$2.43\cdot10^{-3} \pm 9.17\cdot10^{-5}$	$-1.58\cdot10^{-2} \pm 2.62\cdot10^{-2}$	$1.89\cdot10^{-2}$	0.9989	-
Ethion	Soil	5-600	$4.73\cdot10^{-3} \pm 8.29\cdot10^{-5}$	$-6.89\cdot10^{-3} \pm 2.37\cdot10^{-2}$	$1.81\cdot10^{-2}$	0.9997	-8.7
	Solvent calibration	5-600	$5.18\cdot10^{-3} \pm 2.24\cdot10^{-4}$	$-2.80\cdot10^{-2} \pm 6.40\cdot10^{-2}$	$4.61\cdot10^{-2}$	0.9986	-
DDE	Soil	5-600	$3.30\cdot10^{-2} \pm 3.87\cdot10^{-4}$	$5.75\cdot10^{-2} \pm 1.11\cdot10^{-1}$	$8.47\cdot10^{-2}$	0.9999	-25.8
	Solvent calibration	5-600	$4.45\cdot10^{-2} \pm 8.63\cdot10^{-4}$	$-2.84\cdot10^{-2} \pm 2.47\cdot10^{-1}$	$1.78\cdot10^{-1}$	0.9997	-
DDD	Soil	5-600	$3.76\cdot10^{-4} \pm 1.64\cdot10^{-5}$	$-1.37\cdot10^{-3} \pm 4.68\cdot10^{-3}$	$3.58\cdot10^{-3}$	0.9984	-21.8
	Solvent calibration	5-600	$4.81\cdot10^{-4} \pm 1.12\cdot10^{-5}$	$-5.93\cdot10^{-4} \pm 3.21\cdot10^{-3}$	$2.31\cdot10^{-3}$	0.9996	-
Propiconazole	Soil	5-600	$8.17\cdot10^{-3} \pm 2.51\cdot10^{-4}$	$3.99\cdot10^{-1} \pm 7.17\cdot10^{-2}$	$5.49\cdot10^{-2}$	0.9992	-25.0
	Solvent calibration	5-600	$1.09\cdot10^{-2} \pm 5.28\cdot10^{-4}$	$-6.16\cdot10^{-2} \pm 1.51\cdot10^{-1}$	$1.09\cdot10^{-1}$	0.9982	-
Tebuconazole	Soil	5-600	$1.17\cdot10^{-2} \pm 9.70\cdot10^{-5}$	$1.00\cdot10^{-2} \pm 2.78\cdot10^{-2}$	$2.12\cdot10^{-2}$	0.9999	-8.6

	Solvent calibration	5-600	$1.28\cdot10^{-2} \pm 5.74\cdot10^{-4}$	$-7.89\cdot10^{-2} \pm 1.64\cdot10^{-1}$	$1.18\cdot10^{-1}$	0.9984	-
Iprodione	Soil	5-600	$3.17\cdot10^{-4} \pm 1.20\cdot10^{-5}$	$1.29\cdot10^{-3} \pm 3.43\cdot10^{-3}$	$2.62\cdot10^{-3}$	0.9988	20.1
	Solvent calibration	5-600	$2.64\cdot10^{-4} \pm 2.36\cdot10^{-5}$	$-1.92\cdot10^{-3} \pm 6.77\cdot10^{-3}$	$4.87\cdot10^{-3}$	0.9939	-
EPN	Soil	5-600	$4.24\cdot10^{-3} \pm 1.99\cdot10^{-4}$	$-2.07\cdot10^{-2} \pm 5.69\cdot10^{-2}$	$4.36\cdot10^{-2}$	0.9981	-33.1
	Solvent calibration	5-600	$6.34\cdot10^{-3} \pm 4.56\cdot10^{-4}$	$-7.28\cdot10^{-2} \pm 1.31\cdot10^{-1}$	$9.40\cdot10^{-2}$	0.9960	-
Pyriproxyfen	Soil	5-600	$1.59\cdot10^{-2} \pm 2.35\cdot10^{-4}$	$1.80\cdot10^{-1} \pm 6.72\cdot10^{-2}$	$5.14\cdot10^{-2}$	0.9998	-7.0
	Solvent calibration	5-600	$1.71\cdot10^{-2} \pm 6.70\cdot10^{-4}$	$-6.32\cdot10^{-2} \pm 1.92\cdot10^{-1}$	$1.38\cdot10^{-1}$	0.9988	-
DDT	Soil	5-600	$4.90\cdot10^{-2} \pm 6.99\cdot10^{-4}$	$-1.29\cdot10^{-1} \pm 2.00\cdot10^{-1}$	$1.53\cdot10^{-1}$	0.9998	-3.2
	Solvent calibration	5-600	$5.06\cdot10^{-2} \pm 2.00\cdot10^{-3}$	$-2.88\cdot10^{-1} \pm 5.72\cdot10^{-1}$	$4.12\cdot10^{-1}$	0.9988	-
Fenvalerate	Soil	5-600	$1.48\cdot10^{-2} \pm 6.53\cdot10^{-4}$	$-6.98\cdot10^{-2} \pm 1.87\cdot10^{-1}$	$1.43\cdot10^{-1}$	0.9983	80.5
	Solvent calibration	5-600	$8.20\cdot10^{-3} \pm 5.21\cdot10^{-4}$	$-7.06\cdot10^{-2} \pm 1.49\cdot10^{-1}$	$1.07\cdot10^{-1}$	0.9969	-
Esfenvalerate	Soil	5-600	$7.08\cdot10^{-3} \pm 3.80\cdot10^{-4}$	$-8.19\cdot10^{-2} \pm 1.36\cdot10^{-1}$	$9.78\cdot10^{-2}$	0.9970	18.0
	Solvent calibration	5-600	$6.00\cdot10^{-3} \pm 3.50\cdot10^{-4}$	$-1.19\cdot10^{-2} \pm 1.55\cdot10^{-2}$	$8.01\cdot10^{-3}$	0.9927	-
Deltamethrin	Soil	5-600	$9.22\cdot10^{-3} \pm 6.29\cdot10^{-4}$	$-1.09\cdot10^{-1} \pm 1.80\cdot10^{-1}$	$1.30\cdot10^{-1}$	0.9964	-35.1
	Solvent calibration	5-600	$1.42\cdot10^{-2} \pm 6.13\cdot10^{-4}$	$-1.33\cdot10^{-1} \pm 1.76\cdot10^{-1}$	$1.26\cdot10^{-1}$	0.9986	-

b: slope; Sb: standard deviation of the slope; a: intercept; Sa: standard deviation of the intercept; sy/x: standard deviation of the estimate.; R^2: determination coefficient.

Figure S1. Representative MRM chromatograms for five pesticides covering different retention-time regions, obtained from soil samples fortified at the method LOQ (50 μg/kg): (A) propoxur, (B) β-HCH, (C) lindane, (D) aldrin, and (E) DDT. The selected compounds include β-HCH and lindane, which exhibit similar retention times but were successfully distinguished through characteristic MRM transitions. The chromatograms illustrate chromatographic selectivity and analyte detection at the lowest validated concentration level of the method, corresponding to the most demanding validated analytical conditions.

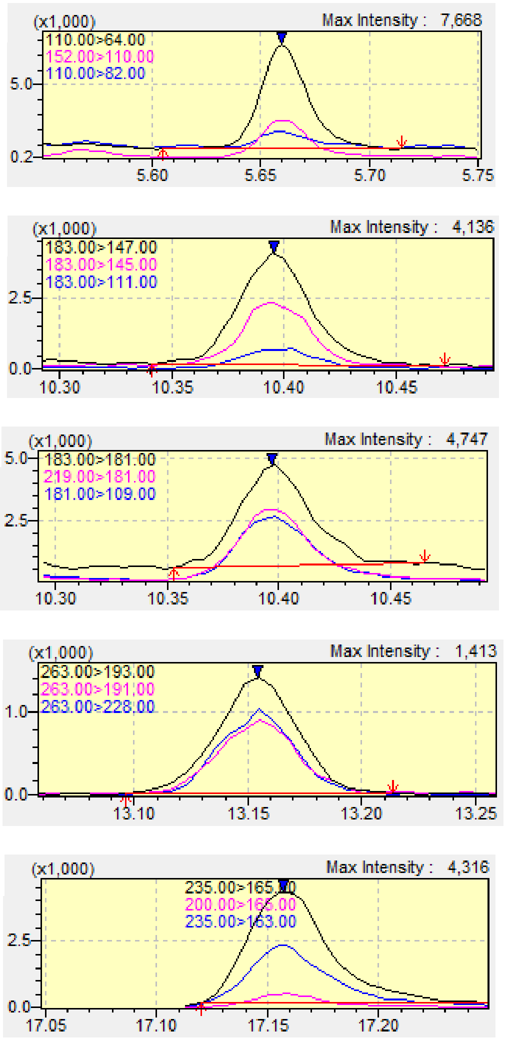

Table S2. Average recoveries (RSDs) of the selected pesticides in the validation sample (n = 5 at each spiking level).

Pesticide	Soil		
	**50 μg/kg**	**200 μg/kg**	**400 μg/kg**
Propoxur	100 (12)	102 (8)	104 (6)
Carbofuran	72 (6)	91 (6)	103 (1)
Diphenylamine	105 (7)	106 (3)	111 (3)
Chlorpropham	112 (3)	104 (2)	105 (4)
Dicloran	98 (10)	99 (2)	103 (4)
Atrazine	98 (6)	101 (1)	96 (4)
Lindane	106 (4)	103 (2)	112 (3)
Propyzamide	104 (13)	100 (3)	99 (7)
β-HCH	108 (3)	104 (3)	105 (2)
α-HCH	108 (2)	103 (3)	106 (2)
Chlorpyrifos-methyl	112 (4)	102 (1)	107 (5)
Parathion-methyl	107 (2)	100 (1)	102 (6)
Alachlor	111 (3)	102 (2)	101 (2)
Tolclofos-methyl	111 (3)	103 (1)	102 (2)
Metalaxyl	**122** (18)	93 (4)	93 (7)
Pirimiphos-methyl	115 (4)	102 (2)	98 (3)
Fenitrothion	118 (2)	101 (2)	97 (7)
Malathion	**121** (4)	102 (2)	97 (5)
Chlorpyrifos	112 (3)	97 (2)	97 (4)
Fenthion	112 (2)	101 (2)	98 (4)
Triadimefon	107 (1)	96 (1)	94 (4)
Aldrin	100 (3)	98 (2)	98 (6)
Pirimiphos-ethyl	**56** (2)	**33** (9)	**32** (7)
Heptachlor	105 (3)	101 (3)	99 (2)
Penconazole	85 (3)	84 (5)	92 (9)
Triflumizole	**168** (16)	100 (6)	103 (7)
Methidathion	111 (18)	101 (3)	98 (8)
Heptachlor *cis*	**123** (14)	100 (4)	99 (3)
Heptachlor *trans*	119 (**21**)	101 (4)	99 (4)
Fenamiphos	85 (16)	70 (16)	**66** (19)
Endosulfan A	101 (3)	102 (2)	101 (3)
Myclobutanil	90 (7)	85 (11)	85 (9)
Dieldrin	106 (4)	99 (5)	98 (4)
Oxyfluorfen	108 (9)	92 (1)	92 (8)
Buprofezin	100 (3)	91 (5)	88 (8)
Endrin	117 (17)	99 (5)	99 (3)
Ethion	99 (17)	87 (6)	84 (4)
DDE	95 (4)	91 (1)	93 (5)
DDD	**126** (17)	97 (5)	104 (4)

Propiconazole	52 (5)	**68** (3)	78 (7)
Tebuconazole	83 (2)	81 (3)	80 (8)
Iprodione	**134** (**35**)	115 (10)	110 (11)
EPN	99 (4)	92 (2)	94 (9)
Pyriproxyfen	81 (20)	86 (4)	84 (8)
DDT	110 (1)	101 (2)	100 (4)
Fenvalerate	120 (20)	95 (4)	88 (6)
Esfenvalerate	**132** (17)	104 (6)	95 (11)
Deltamethrin	106 (19)	93 (3)	89 (8)

Bold text indicates recovery outside the 70-120% range and RSDs > 20 %.
